# Computational Study of Dipole Radiation in Re‐Absorbing Perovskite Semiconductors for Optoelectronics

**DOI:** 10.1002/advs.202003559

**Published:** 2021-01-04

**Authors:** Changsoon Cho, Neil C. Greenham

**Affiliations:** ^1^ Dresden Integrated Center for Applied Physics and Photonic Materials (IAPP) Technische Universität Dresden Dresden 01187 Germany; ^2^ Cavendish Laboratory Department of Physics University of Cambridge J.J. Thomson Avenue Cambridge CB3 0HE UK

**Keywords:** light‐emitting diodes, microcavities, optical modelling, perovskites, photon recycling, Stokes shift, transfer‐matrix formalism

## Abstract

Compared to organic emitters, perovskite materials generally have a small Stokes shift and correspondingly large re‐absorption of dipole emission. Classical optical modelling methods ignoring re‐absorption do not provide an adequate description of the observed light emission properties. Here, optical modelling methods and design rules for perovskite light‐emitting diodes are presented. The transfer‐matrix formalism is used to quantify the Poynting vectors generated by a dipole radiating inside a perovskite optoelectronic device. A strategy is presented to deal with non‐radiative coupling to nearby emissive material that can otherwise lead to non‐physical divergence in the calculation. Stability issues are also investigated regarding coherence of the light propagating in the substrate and the absence of a light absorber in the system. The benefit of the photon recycling effect is taken into account by recursive calculation of the dipole generation profile. The simulation results predict that a high external quantum efficiency of ≈40% is achievable in formamidinium lead triiodide‐based perovskite light‐emitting diodes, by optimization of microcavity, dipole orientation, and photon recycling effects. Contrary to conventional device structures currently reported, this work highlights the benefits of thick charge transport layers and thick perovskite with small Stokes shift.

## Graphical abstract

The methodology for optical modelling of perovskite light‐emitting diodes is investigated. Reabsorption in the perovskite causes divergence in classical methods of optical modelling. Here, the divergence is resolved by exploring the physical meaning of non‐radiative nearfield coupling to nearby emissive material. This enables the quantitative analysis of light emission in perovskites, including the contribution of photon recycling.

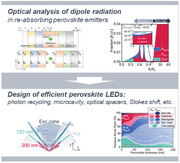

## 1 Introduction

Perovskite light‐emitting diodes (LEDs) are currently receiving great attention in the field of lighting and displays, exhibiting low‐cost processibility, narrow emission spectrum, bandgap tunability, and high photoluminescence (PL) quantum efficiency. Since the demonstration of 0.1% external quantum efficiency (EQE) in 2014,^[^ ^1^ ^]^ perovskite LEDs have shown rapid growth in efficiencies and have broken the EQE barrier of 20% in 2018,^[^ ^2^ ^]^ comparable to the efficiency of organic LEDs. While perovskite LEDs have been mostly studied with the existing design principles established in the field of organic LEDs, it has been recently reported that the distinct optical properties of perovskite such as photon recycling play an important role in their performance.^[^ ^3^ ^]^

Accurate optical modelling of LEDs is an important issue not only for scientific studies, but also for rapid optimization of device design in industry. While various simulation methods have been extensively studied in the field of organic LEDs,^[^ ^4^ ^]^ they are mostly based on the assumption of a non‐absorbing emissive layer, which is a good approximation for most organic emitters, but not applicable to perovskite emitters having a large overlap between absorption and emission spectra (i.e., small Stokes shift). When a dipole radiates in an absorptive medium, strong coupling of the dipole with nearby material causes a divergence of conventional calculations, leading to a prediction of zero outcoupling.^[^ ^5^ ^]^ Moreover, those models neglecting emitter absorption miss both the loss from reabsorption and the benefit from photon recycling.

Here, we present an optical model that can be applied in general to re‐absorbing emissive systems. We resolve the divergence issue in the modelling by noting that non‐radiative near‐field coupling essentially reproduces the emissive state a negligible distance away, and hence its physical effects can be ignored. This makes it possible to incorporate the effects of photon recycling in a self‐consistent manner. The proposed method was previously utilized in our recent work and successfully supported the experimental investigation of photon recycling effects in green‐emitting (PEA)_2_Cs*_n_*_‐1_Pb*_n_*Br_3_ *_n_*_+1_ perovskite LEDs.^[^ ^3c^ ^]^ Here, we focus on general optical aspects of perovskite emitters and explain the modelling methodology in more detail. Then, we apply it to LEDs based on the infrared‐emitting formamidinium lead triiodide (FAPbI_3_) perovskite, to establish general design rules for perovskite LEDs. We analyze the role of microcavity effects, dipole orientation, and photon recycling on optical outcoupling for different device structures and internal radiation efficiencies, allowing us to propose optimized structures.

## 2 Calculation of Power Fluxes

**Figure 1:**
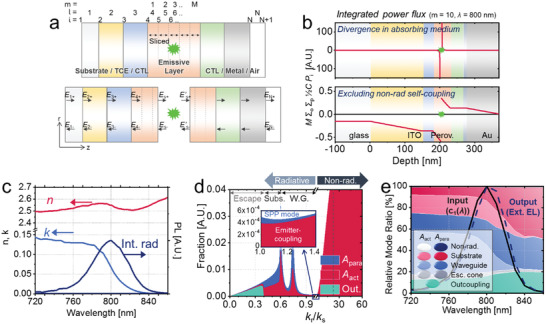
a) A multilayer thin‐film LED structure, consisting of *N* layers (*l*) comprising substrate, transparent conductive electrode (TCE), charge transporting layers (CTLs), emissive layer (e.g., perovskite), and metal electrode, having *N*+1 interfaces (*i*). The emissive layer is sliced into *M* layers and dipoles are distributed over the slices. The optical electric fields at each interface consist of *E_i_*_+_ and *E_i_*_‐_ components with opposite propagation directions along the *z*‐axis. b) Power flux (= *M Σ_o_ Σ_p_* ½*C P*_i_, for a dipole at *m* = 10 and *λ* = 800 nm) at each interface in the devices including (top) and excluding (bottom) non‐radiative emitter‐coupling in the calculation (integrated over 0 ≤ *k_r_*/*k_s_* ≤ 100). c) Refractive index (*N* = *n*+*jκ*) and internal radiation spectrum of FAPbI_3_ perovskite used in the simulation. d) Relative amounts of dipole energy (= *S_zi_ k_r_* d*k_r_* / *N_s_ k_zs_* ^2^ at *m* = 10 and *λ* = 800 nm) which are outcoupled, re‐absorbed by emitter (*A*_act_), or re‐absorbed by parasitic layers (*A*_para_), as a function of relative radial propagation vector *k_r_*/*k_s_*. For *k_r_* > *k_s_*, the dipole is non‐radiatively coupled with nearby parasitic absorbers (surface plasmon polariton (SPP) mode) or the emitter itself. e) Fraction of photon propagation in various modes: outcoupling, re‐absorption within escape cone, waveguide trapping, substrate trapping, and non‐radiative parasitic dissipation (SPP), as a function of wavelength. The non‐outcoupled radiative modes are split into *A*_act_ and *A*_para_, depending on the final destination of photons. Due to the spectral dependence of the outcoupling mode (mainly caused by the larger perovskite absorption for shorter wavelengths), the calculated external EL spectrum (dashed line) is slightly red‐shifted compared to the internal spectrum.

**Figure** **1**a depicts a generalized multilayer structure, consisting of a transparent conductive electrode, charge transport layers, an emissive layer, and a metal electrode. The emissive layer is divided uniformly into *M* slices, indexed by *m* = 1 … *M*. Each of the *N* layers in the overall structure is labelled by the index *l*, now treating each of the *M* slices of the emissive layer as a separate layer, as shown. The *N*+1 interfaces between these layers are labelled by the index *i*. *E_i_*_+_ and *E_i_*_−_ indicate the amplitudes of forward‐going and backward‐going electric fields (*E*‐fields) right after each interface *i*, respectively, induced by radiation. It is well known that the relationship between those *E*‐field vectors can be calculated using transfer‐matrices^[^ ^6^ ^]^:

(1) $$\left(\begin{array}{c} E_{i+}\\ E_{i-} \end{array}\right)=L_{i}I_{i+1}\left(\begin{array}{c} E_{\left(i+1\right)+}\\ E_{\left(i+1\right)-} \end{array}\right)$$

The phase matrix *L_i_* is described by

(2) $$L_{i}=\;\left[\begin{array}{cc} \exp\left(-jk_{zi}d_{i}\right) & 0\\ 0 & \exp\left(jk_{zi}d_{i}\right) \end{array}\right]$$

where *k_zi_* is a complex *z*‐wavevector obeying *k_zi_* ^2^ + *k_r_* ^2^ = *k_i_* ^2^ = (2*πN*_i_/*λ*)^2^, *k_r_* is the radial wavevector, *k_i_* is the total wavevector, *N_i_* is a refractive index of *n_i_*+*jκ_i_*, and *d_i_* is a thickness at *i*‐th layer. (*λ*: wavelength) The refractive matrix *I_i_* can be obtained from Fresnel transmission (*t_i_*) and reflection (*r_i_*) coefficients for *E*‐fields:

(3) $$I_{i}=\;\left[\begin{array}{cc} 1/t_{i} & r_{i}/t_{i}\\ r_{i}/t_{i} & 1/t_{i} \end{array}\right]$$

where

(4) $$\begin{array}{ccc} t_{i} & = & \left\{\begin{array}{c} \frac{2k_{\mathrm{zi}}}{k_{\mathrm{zi}}+k_{z\left(i+1\right)}}\;\left(\mathrm{TE}\;\mathrm{mode}\right)\\ \frac{2k_{\mathrm{zi}}N_{i}N_{i+1}}{k_{\mathrm{zi}}N_{i+1}^{2}+k_{z\left(i+1\right)}N_{i}^{2}}\;\left(\mathrm{TM}\;\;\mathrm{mode}\right. \end{array}\right)\;\;\mathrm{and}\\ r_{i} & = & \left\{\begin{array}{c} \frac{k_{\mathrm{zi}}-k_{z\left(i+1\right)}}{k_{\mathrm{zi}}+k_{z\left(i+1\right)}}\;\left(\mathrm{TE}\;\;\mathrm{mode}\right)\\ \frac{-k_{\mathrm{zi}}N_{i+1}^{2}+k_{z\left(i+1\right)}N_{i}^{2}}{k_{\mathrm{zi}}N_{i+1}^{2}+k_{z\left(i+1\right)}N_{i}^{2}}\;\left(\mathrm{TM}\;\mathrm{mode}\right) \end{array}\right. \end{array}$$

To treat emission from dipoles within this structure, we follow the approach of Benisty et al.^[^ ^4a^ ^]^ When a dipole is positioned at the centre of *m*‐th emitting slice, we add an additional interface *s* at this position and define (*E_s_*_+_, *E_s_*_‐_) and (*E*′*_s_*_+_, *E*′*_s_*_‐_) to be the *E*‐field vectors immediately before and after the dipole, respectively, as shown in Figure 1a. Then, the difference between those two vectors is fixed by the source terms of the dipole:

(5) $$\left(\begin{array}{c} A_{+}\\ A_{-} \end{array}\right)=\;\left(\begin{array}{c} {E^{\prime }}_{s+}\\ {E^{\prime }}_{s-} \end{array}\right)-\;\left(\begin{array}{c} E_{s+}\\ E_{s-} \end{array}\right)$$

Here, normalized source terms *A*_+_ and *A*_‐_ were used as shown in **Table** **1**, and *E*‐fields become unitless. If the first and last layers are infinitely thick and there is no external light source, we can set *E*_1+_ = 0 and *E_N_*_‐_ = 0 as boundary conditions. Then, by combining Equations (1) and (5),

(6) $$\begin{array}{ccc} \left(\begin{array}{c} A_{+}\\ A_{-} \end{array}\right) & = & L_{s}I_{n+1}\left(\prod_{i\;=\;n+1}^{N-1}L_{i}I_{i+1}\right)\left(\begin{array}{c} E_{N+}\\ 0 \end{array}\right)\\ & & -\;{\left\{\left(\prod_{i\;=\;1}^{n-1}L_{i}I_{i+1}\right)L_{s}\right\}}^{-1}\left(\begin{array}{c} 0\\ E_{1-} \end{array}\right) \end{array}$$

where the *n*‐th layer contains the dipole and *L_s_* represents the phase matrix for the half propagation in this layer. Equation (6) provides two equations for two unknown variables of *E_N_*_+_ and *E*_1‐_. Hence, we can obtain all the *E*‐field vectors inside the structure for given dipoles by solving Equations (1)–(6). Then, at each interface, z‐directional Poynting vectors can be calculated from the *E*‐field vectors:^[^ ^7^ ^]^

(7) $$S_{\mathrm{zi}}=\left\{\begin{array}{c} \mathrm{Real}\left\{N_{i}\cos\varphi_{i}{\left(E_{i+}+E_{i-}\right)}^{*}\left(E_{i+}-E_{i-}\right)\right\}\left(\mathrm{TE}\;\;\mathrm{mode}\right)\\ \mathrm{Real}\left\{N_{i}\cos{\varphi_{i}}^{*}{\left(E_{i+}+E_{i-}\right)}^{*}\left(E_{i+}-E_{i-}\right)\right\}\left(\mathrm{TM}\;\mathrm{mode}\right) \end{array}\right.\;$$

**Table 1:** Normalized source terms of dipoles for various polarizations and orientations. Reproduced from previous literature.^[^ ^8^, ^12^ ^]^ *φ_s_* indicates the propagation angle inside the emissive layer. TE‐polarized vertical dipoles are ignored as they are not distinguishable from TM‐polarized vertical dipoles

	TE‐polarized	TM‐polarized
Horizontal	$\left[\begin{array}{c} A_{+}\\ A_{-} \end{array}\right]=\sqrt{\frac{3}{16\pi }}\left[\begin{array}{c} -1\\ 1 \end{array}\right]$	$\left[\begin{array}{c} A_{+}\\ A_{-} \end{array}\right]=\sqrt{\frac{3}{16\pi }}\cos\varphi_{s}\left[\begin{array}{c} -1\\ 1 \end{array}\right]$
Vertical	$\left[\begin{array}{c} A_{+}\\ A_{-} \end{array}\right]=\left[\begin{array}{c} 0\\ 0 \end{array}\right]$	$\left[\begin{array}{c} A_{+}\\ A_{-} \end{array}\right]=\sqrt{\frac{3}{8\pi }}\sin\varphi_{s}\left[\begin{array}{c} 1\\ 1 \end{array}\right]$

Note that some constants of proportionality are ignored in this equation for simplicity. The propagation angle *φ_i_* is determined for a given radial propagation vector *k_r_* as

(8) $$\cos\;\varphi_{i}=\frac{k_{zi}}{k_{i}}\;\;=\;\sqrt{1-\frac{k_{r}^{2}}{{\left(2\pi N_{i}/\lambda \right)}^{2}}}\;$$

Then, the relative power fluxes, normalized to those for the free space dipole, can be obtained by integration over *k*_r_:

(9) $$P_{i}=\int_{0}^{\infty }\frac{2\pi \;}{N_{s}k_{zs}^{2}}\;S_{zi}\;k_{r}dk_{r}\;$$

At each interface, the magnitudes of the *E*‐fields and Poynting vectors depend on the various dipole properties such as the emission angle (i.e., relative *k_r_*), wavelength (*λ*), position (*m* in Figure 1a), and orientation (*o =* vertical (*z*) or horizontal (*x*,*y*)), and on the polarization (*p =* TE or TM). By assuming that all dipoles can be represented by the sum of the three orthogonal dipoles, choices for *o* can be narrowed to two—vertical (*z*) or horizontal (*x*,*y*). We define the distribution of dipole emission properties by the weighting factor *C*(*λ*,*m*,*o*) and then integrate over orientation, position and polarization to obtain the spectra of light extraction efficiency (LEE) and absorption (Abs) of each layer:

(10) $$\;\mathrm{LEE}\left(\lambda \right)\;=\frac{\sum_{m}\sum_{o}\sum_{p}\frac{1}{2}\left[C\left(\lambda ,m,o\right)\left(-P_{1}\left(\lambda ,\;m,o,p\right)+P_{N}\left(\lambda ,m,o,p\right)\right)\right]}{\sum_{m}\sum_{o}\sum_{p}\frac{1}{2}\left[C\left(\lambda ,m,o\right)\left({P^{\prime }}_{s}\left(\lambda ,m,o,p\right)-P_{s}\left(\lambda ,m,o,p\right)\right)\right]}$$

(11) $$\;\mathrm{Ab}s_{l}\left(\lambda \right)\;=\left\{\begin{array}{c} 0\;\;\;\;\;\;\;\;\;\left(\mathrm{for}\;\mathrm{the}\;\mathrm{layer}\;\mathrm{containing}\;\;\mathrm{the}\;\mathrm{dipole}\right)\\ \frac{\sum_{m}\sum_{o}\sum_{p}\frac{1}{2}\left[C\left(\lambda ,m,o\right)\left(P_{l}\left(\lambda ,m,o,p\right)-P_{l+1}\left(\lambda ,m,o,p\right)\right)\right]}{\sum_{m}\sum_{o}\sum_{p}\frac{1}{2}\left[C\left(\lambda ,m,o\right)\left({P^{\prime }}_{s}\left(\lambda ,m,o,p\right)-P_{s}\left(\lambda ,m,o,p\right)\right)\right]}\;\left(\mathrm{for}\;\mathrm{other}\;\mathrm{layers}\right) \end{array}\right.$$

*Σ_p_* denotes the sum of the values for TE and TM polarization (*p*) modes. It should be noted that backward power fluxes have negative values. The absorption of the slice containing the dipole is set to zero to avoid divergence, as described in the next section. $\sum_{m}\sum_{o}\sum_{p}\frac{1}{2}\left[C\left(\lambda ,m,o\right)\left({P^{\prime }}_{s}\left(\lambda ,m,o,p\right)-P_{s}\left(\lambda ,m,o,p\right)\right)\right]$ indicates the relative dipole source power (*S*_0_(*λ*)), increased or decreased by the optical resonance, where *P_s_* and *P_s_*’ are calculated from (*E_s_*_+_, *E_s_*_‐_) and (*E’_s_*_+_, *E’_s_*_‐_), respectively. In this manuscript, we assume a uniform distribution of dipoles over emitting position, and orientation by inputting:

(12) $$\begin{array}{ccc} C\;\left(\lambda ,m,o\right) & = & c_{1}\;\left(\lambda \right)\cdot c_{2}\left(m\right)\cdot c_{3}\;\left(o\right)\\ & = & \left\{\begin{array}{c} c_{1}\left(\lambda \right)\cdot \;\frac{1}{M}\cdot \;\frac{1}{3}\;\;\left(\mathrm{vertical}\right)\\ c_{1}\left(\lambda \right)\cdot \frac{1}{M}\cdot \;\frac{2}{3}\;\;\left(\mathrm{horizontal}\right) \end{array}\right.\; \end{array}$$

where *c_1_*(*λ*) is the internal radiation spectrum of a given emitter, satisfying

(13) $$\int_{0}^{\infty }\sum_{m}\sum_{o}C\left(\lambda ,m,o\right)\;d\lambda =\int_{0}^{\infty }c_{1}\left(\lambda \right)\;d\lambda \;=\;1$$

Although we have assumed a uniform initial spatial distribution of emitting dipoles, in future work it would be possible to incorporate a more precise distribution, for example that predicted by a drift‐diffusion model.^[^ ^7^, ^8^ ^]^

## 3 Difficulties in Modelling for Reabsorbing Emissive Media

While various optical modelling methods have been used in the field of OLEDs,^[^ ^4^ ^]^ modelling systems containing re‐absorbing emitters such as perovskites has been a challenging problem.^[^ ^5^ ^]^ The first difficulty arises from the complex variables. The equations in the previous section are mostly based on the assumption that the refractive index of the source layer is of purely real. Using a complex refractive index for the source layer (*N_s_* = *n_s_*+*jκ_s_*, *κ_s_* > 0) induces complex variables such as source propagation angle (*φ*_s_) and power flux. Such complexity can be simply resolved by setting the imaginary part of the refractive index to be zero in an ultrathin slice containing the dipole. For example, among the *M* slices in Figure 1a, we assume that the slice containing the dipole is non‐absorbing while the rest retain non‐zero *κ*, as outlined in Equation 11. Although not technically Kramers‐Kronig consistent, this approach is found to have a negligible effect on the overall propagation of light in the system.

However, even with the assumption of a non‐absorbing emissive slice, there still remains another challenge of divergence coming from nearby absorbing slices. The top image of Figure 1b shows the calculated power flux inside a perovskite LED, consisting of glass/ indium tin oxide (ITO, 150 nm)/ ZnO (30 nm)/ FAPbI_3_ perovskite (50 nm)/ poly(9,9‐dioctyl‐fluorene‐*co*‐*N*‐(4‐butylphenyl)diphenylamine) (TFB, 40 nm)/ MoO_3_ (7 nm)/ Au (100 nm),^[^ ^2b^ ^]^ at the single wavelength of 800 nm. The refractive index (*n*+*jκ*) of the perovskite shown in Figure 1c was obtained by fitting the calculation to the transmission and reflection measured for a FAPbI_3_ perovskite film.^[^ ^9^ ^]^ *κ* in the band‐edge region was fine‐tuned by assuming that the measured PL spectrum follows the spectrum of black‐body radiation multiplied by the absorption of the film, as done elsewhere.^[^ ^10^ ^]^ The internal radiation spectrum (c_1_(*λ*)) was obtained by dividing the measured external PL(*λ*) by the calculated LEE(*λ*) and normalizing it. The refractive indices of glass and TFB were assumed to be 1.5 and 1.57, respectively, and those for other layers were obtained from the literature.^[^ ^11^ ^]^ The emitter was divided into 20 slices (*M* = 20, each is 2.5 nm thick), and the dipole was positioned at *m* = 10, in a slice that was assumed to be non‐absorbing as explained above. As shown in the image, while the source power diverges (>> 100) at the emitting position, much larger than the value of 1 corresponding to free space, it drops rapidly with distance and only 0.151 is outcoupled. The fraction of radiated power coupled out thus tends toward zero.

This apparently unphysical prediction from the classical calculation can be attributed to the non‐radiative coupling of the dipole with nearby absorbing slices. The integrated outward radiation of a sphere (radius *R*) containing a Hertzian dipole is known to be:^[^ ^3^, ^12^ ^]^

(14) $$\begin{array}{c} P\left(R\right)\;\approx \;\left(\;2\alpha \beta R^{-3}\;+4\alpha^{2}\beta R^{-2}\;+2\alpha \beta \left(\;\alpha^{2}\;+\beta^{2}\;\right)R^{-1}\;+\beta {\left(\;\alpha^{2}\;+\beta^{2}\;\right)}^{2}\;\right)e^{-2\alpha R} \end{array}$$

where *β*−*jα* represents a propagation constant. While *P*(*R*) is independent of *R* in a non‐absorbing medium (*α* = 0), terms scaling as *R* ^–1^, *R* ^–2^, and *R* ^–3^ cause divergence for small *R* in case that the medium is dispersive (*α* ≠ 0).^[^ ^3c^ ^]^ Therefore, the dipole energy is rapidly dissipated in the near‐field region through non‐radiative pathways.

Figure 1d shows the relative fraction of the dipole emission as a function of the in‐plane wavevector, *k_r_* normalized to *k_s_* ( = 2*πN_s_*/*λ*). Our Poynting‐vector based calculation allows us to separate the non‐outcoupled modes into those eventually re‐absorbed by emitter (*A*_act_) and parasitic layers (*A*_para_) such as the ITO and the metal electrode. To make a photon propagate in a layer *l*, the *z*‐wavevector *k_zl_* (*k_zl_* ^2^ = (2*πN_l_*/*λ*)^2^−*k_r_* ^2^) should have a real component in the layer. Hence, the escape cone, substrate mode, and waveguide mode are formed in the range of [*k_r_* < 2*πN*_air_/*λ*], [2*πN*_air_/*λ < k_r_* < 2*πN*_glass_/*λ*], and [2*πN*_glass_/*λ < k_r_* < 2*πN_s_*/*λ*], respectively, as shown in Figure 1d. While photons cannot propagate for *k_r_* > 2*πN_l_*/*λ* (i.e., *k_zl_* ^2^ < 0) in non‐absorbing layers, for dispersive layers having a complex refractive index (*N_l_* = *n_l_*+*jκ_l_, κ_l_* > 0), *k_zl_* can have a real component and a propagation mode appears even for large *k_r_*. In OLEDs, this type of non‐radiative mode for large *k_r_* is mostly associated with electrodes, and is known as a surface plasmon polariton (SPP) mode. However, for emitters that are re‐absorbing, such modes can appear even in the nearby slices of the emitter layer, with a coupling strength that diverges as the mesh size decreases, since this mode is inversely proportional to the fourth power of the dipole‐absorber distance (from point dipole to planar absorber).^[^ ^3c^ ^]^ Figure 1d shows the mode fraction for *k_r_* > *k_s_* greatly dominates other modes and it is mostly caused by the coupling with a perovskite absorber (*A*_act_), rather than coupling with parasitic layers (*A*_para_, SPP mode).

## 4 Investigation of Non‐Radiative Energy Transfer

The result shown above, predicting zero outcoupling, is obviously not true in the real world. Therefore, the experimentally reported nonzero emission of perovskite emitters provides strong evidence that dipole energy coupled back into the emitter layer is not lost from the system. If the emitter‐coupled energy can generate a new dipole at the re‐absorbed position, it can be considered as a simple energy transfer process, which should not affect the outcoupling efficiency. While photon recycling for radiative modes will be discussed later, here we exclude non‐radiative coupling to the emitter from the calculation by modifying Equation (9):

(15) $$\begin{array}{c} P_{i}=\left\{\begin{array}{c} \int_{0}^{k_{s}}\frac{2\pi \;}{N_{s}k_{\mathrm{zs}}^{2}}\;S_{\mathrm{zi}}\;k_{r}dk_{r}\;\left(\mathrm{interfaces}\;\mathrm{between}\;\mathrm{emissive}\;\mathrm{meshes}\right)\\ \int_{0}^{\infty }\frac{2\pi \;}{N_{s}k_{\mathrm{zs}}^{2}}\;S_{\mathrm{zi}}\;k_{r}dk_{r}\;\left(\mathrm{other}\;\mathrm{interfaces}\right) \end{array}\right. \end{array}$$

The exclusion of that mode implies the recycling of non‐radiatively emitter‐coupled photons with 100% efficiency. This assumption can be rationalized by the fact that such near‐field coupling occurs very fast, with a rate boosted by its diverging mode intensity (i.e., diverging Purcell factor^[^ ^3^, ^13^ ^]^), which dominates any possible optical loss having finite rate. In the picture of semiconductor physics, this non‐radiative near‐field coupling can be understood as an optical expression of Förster resonance energy transfer.^[^ ^3^, ^14^ ^]^ The slight spatial translation of dipoles from the non‐radiative near‐field coupling is not taken into account in the calculation.

The bottom image of Figure 1b shows the re‐calculated power flux profile according to Equation (16). The source power is reduced to 0.927, and the outcoupling efficiency is 0.151/0.927 = 16.3%. Figure 1e shows the calculated mode fractions as a function of wavelength. Since the extinction coefficient of the perovskite is larger for short wavelengths, the short‐wavelength region tends to have higher perovskite re‐absorption (*A*_act_) and lower outcoupling efficiency. Hence, the spectrum of external radiation is red‐shifted compared to the internal luminescence (*c*_1_(*λ*)) we assumed.

## 5 Stability of Calculation

As shown in Equations (9) and (16), power flux is obtained by integrating Poynting vectors over *k_r_* increasing from 0 to infinity. When non‐radiative emitter‐coupling is excluded, the Poynting vector for large *k_r_* (i.e., SPP mode) rapidly tends to zero (Figure 1d) and we can terminate the integration at a certain value of *k_r_*. The accuracy of the calculation is determined by the resolution of the *k_r_* sweep. For layers more than one order thicker than the wavelength (e.g., the substrate), a small change of *k_r_* causes large changes in *E*‐field phase (Equation (2)) after propagation through the layer. Therefore, to compensate for such sensitive fluctuation of the result, the calculation requires a very precise sweep of *k_r_*, which lengthens the running time excessively.

**Figure 2:**
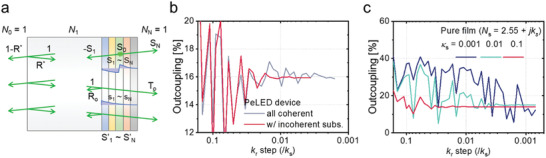
a) Recursive photon circulation in an LED, removing coherence in the substrate (white region). When a photon is emitted toward the substrate (having a Poynting vector of ‐*S*_1_), it is partially reflected at the top surface (Fresnel reflection of *R* ^*^) and returned toward the multilayer. The photons reflected at the multilayer (fraction of *R_o_*) meet the top surface again and repeat the circulation until all energy is outcoupled or dissipated. The final Poynting vectors at the interfaces are represented by the sum of the initially generated ones (*S*_1_ ≈ *S_N_* from coherent calculation in the multilayer structure) and those for all the recursive processes (*s*_1_ ≈ *s_N_* for the photons impinging from substrate to the multilayer). b) Calculated direct outcoupling ratio of photons emitted in a perovskite LED, having a structure of glass (1 mm)/ ITO (150 nm)/ ZnO (30 nm)/ FAPbI_3_ perovskite (50 nm)/ TFB (40 nm)/ MoO_3_ (7 nm)/ Au (100 nm), for the calculations using various *k*_r_ resolutions, represented by *k_r_* step over *k_s_*. Compared to the all coherent calculations, the calculation removing coherence in the substrate is easily stabilized even with a large *k_r_* step. c) Monochromatically (*λ* = 800 nm) calculated direct outcoupling ratio of photons emitted in a perovskite film, having a structure of glass (incoherent)/ perovskite (50 nm), assuming a complex perovskite refractive index of *N_s_* = 2.55 + *jκ_s_*. The result is more easily stabilized with larger extinction coefficient of the film (*κ_s_*), in the absence of any other absorbing layers

Removing optical coherence in thick layers such as the substrate is an efficient way to achieve high accuracy with reduced running time.^[^ ^15^ ^]^ **Figure** **2**a represents a device ignoring coherence in the substrate. When a dipole radiates inside the emissive layer, part of the radiation propagates through the substrate with a Poynting vector magnitude of ‐S_1_, which is calculated from Equations (1)–(8) assuming an infinitely thick substrate. When it reaches the top interface to the air (*N*_0_ = 1), a small fraction ($R^{*}=|\frac{N_{1}-N_{0}}{N_{1}+N_{0}}{|}^{2}\;$) of it is reflected toward the multilayer by Fresnel reflection. For the reflected light impinging on the multilayer, the transmission (*T*_o_), reflection (*R*_o_), and absorption of each layer can be obtained by calculating relative Poynting vectors (*s*_1_—*s_N_*, normalized to the incident light) via TMF for an external light source.^[^ ^6^, ^15^ ^]^ The reflected light can be either outcoupled or reflected back again at the top surface. When such internal reflection occurs infinitely, the Poynting vector at each layer is updated to

(16) $$\begin{array}{ccc} {S^{\prime }}_{i} & = & S_{i}+\left(-S_{1}\right)\times R^{*}\times s_{i}+\left(-S_{1}\right)\times R^{*}\times R_{o}\times R^{*}\times s_{i}+..\\ & = & S_{i}-\frac{S_{1}R^{*}s_{i}}{1-R^{*}R_{o}} \end{array}$$

which can be used instead of *S_i_*. For example, the forward‐direction outcoupling efficiency can be expressed as:

(17) $$\mathrm{LE}{E^{\prime }}_{f}=\;\mathrm{LE}E_{f}-\;\frac{\mathrm{LE}E_{f}\times R^{*}\left(1-R_{o}\right)}{1-R^{*}R_{o}}=\mathrm{LE}E_{f}\times \frac{1-R^{*}}{1-R^{*}R_{o}}$$

Figure 2b shows the calculated LEE of the perovskite LED with Equations (17) and (18), compared to the coherent calculation assuming a 1 mm‐thick substrate, as a function of the *k_r_* step size relative to *k_s_*. While both calculations converge to the same outcoupling of 15.9%, the removal of coherence is shown to stabilize the calculation with a much larger step size (i.e., lower resolution) for *k_r_*. For all calculations in this manuscript except for Figure 2, we ignored coherence in the substrate and used a *k_r_* step of 0.0056 *k_s_*, which divides the dipole radiative mode into 180 slices of *k_r_*, and achieves a relative error below 0.1%.

Although reabsorption within the emissive layer causes complications as described above, it is necessary to have some absorption in the modelled structure in order to achieve stability in the TMF calculation. In a device, the electrodes typically provide sufficient broad‐band absorption to avoid this problem. In a simple film, however, care must be taken, particularly at longer wavelengths where the absorption is small. For example, Figure 2c shows the monochromatically calculated LEE of a pure film,^[^ ^3c^ ^]^ having a complex refractive index of *N_s_* = 2.55 + *jκ_s_* at *λ* = 800 nm, as a function of the *k_r_* step size used in the calculation. As the extinction coefficient *κ_s_* decreases, the resolution required for stability is found to rapidly increase; the result for *κ_s_* = 0.001 is not stabilized even for a *k_r_* step size as small as 10^–3^ *k_s_*. Stability must therefore be monitored carefully in systems where absorption is low.

## 6 Photon Recycling Effect

**Figure 3:**
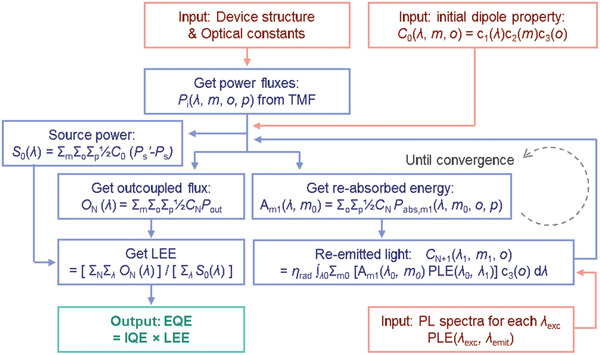
Algorithm for the outcoupling efficiency calculation, taking photon recycling into account. When a dipole radiates, outcoupled power fluxes are recursively calculated from the photons re‐emitted with a renewed spectrum (PLE(*λ*_0_ *, λ*_1_)) at the re‐absorbed position (*m*_1_), and integrated over the various initial dipole positions (*m*), orientations (*o*), for both polarizations (*p*). LEE is calculated by dividing the total output flux by the source power, and multiplied by IQE to get EQE.

In re‐absorbing emitters such as perovskites, emitted photons can be re‐absorbed in the active layer and subsequently re‐radiated.^[^ ^3b^ ^]^ We note that this “photon recycling” process is distinct from the non‐radiative coupling discussed above. If the re‐radiation efficiency is high, photon recycling can be beneficial in achieving outcoupling of photons in trapped modes. Re‐emission randomizes the direction of emission, so recursive recycling events can push the outcoupling limit toward 100%.^[^ ^3a,3c,3d^ ^]^ Therefore, quantification of photon recycling is key to accurately simulating the performance of optoelectronic devices based on materials with small Stokes shifts. Here, we describe in detail a methodology to achieve this, as outlined in **Figure** **3**.

First, power fluxes *P_i_*(*λ*, *m*, *o*, *p*) are calculated from Equation (16). Then, we can obtain the source power (*S*_0_(*λ*)), output flux (*O*(*λ*)), and re‐absorbed energy of each emitter slice (*A*_m1_(*λ*, *m*_0_)), using an input dipole property of *C*_0_(*λ*, *m*, *o*), where *A*_m1_(*λ*, *m*_0_) indicates the energy absorbed by a slice *m*_1_, for the dipole radiation in a slice *m*_0_. The absorbed energy is re‐radiated in the absorbing slice with an efficiency *η*_rad_, ignoring diffusion of charge carriers or excitons. For the re‐radiating dipole, the orientation (*c*_3_(*o*)) is assumed to be re‐randomized, and hence independent of the original dipole orientation. For most perovskites the emission spectrum is independent of excitation wavelength, so a single emission spectrum can be used.^[^ ^3^, ^16^ ^]^ In materials where there is strong inhomogeneous broadening and incomplete relaxation, the excitation wavelength dependence of the emission spectrum (PLE(*λ*_exc_ *, λ*_emit_)) can be directly measured and used as an input to the calculation. The calculated properties of the re‐emitting dipoles are then recursively used as an input to get the next *O*_N_(*λ*) and *A*_m1_(*λ*, *m*_0_), until no energy remains. Then the total output flux is obtained by summing *O*_N_(*λ*), and LEE can be calculated by dividing it by *S*_0_. The EQE can be obtained by multiplying internal quantum efficiency (IQE) by LEE, where IQE = *η*_inj_ × *η*_rad_ and *η*_inj_ represents the number of initial recombination events occurring per charge carrier flowing in the external circuit.^[^ ^3^, ^17^ ^]^ We note that when photon recycling is included *η*_rad_ affects not only IQE, but also LEE. In the simulations shown later we assume a constant value of *η*_rad_,_,_ but this assumption could in general be relaxed to allow *η*_rad_ to vary through the thickness of the emissive layer, for example to allow for variation in the radiative rate due to cavity effects, or variation in the non‐radiative rate due to the presence of quenching sites.

## 7 Optical Investigation for Efficient Outcoupling

**Figure 4:**
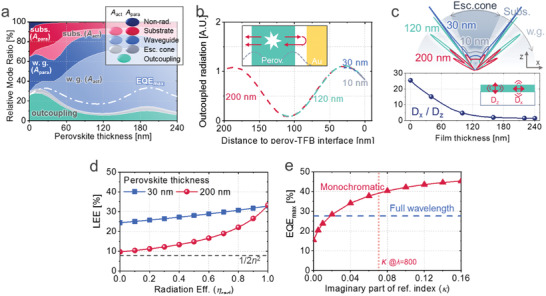
Simulation results for FAPbI_3_‐based perovskite LEDs. a) Relative ratio of the photon propagation through various modes of outcoupling, re‐absorption within escape cone, waveguide trapping, substrate trapping, and non‐radiative parasitic dissipation (SPP), as a function of perovskite thickness. The non‐outcoupled radiative modes are split into *A*_act_ and *A*_para_, depending on the final destination of photons. The white dashed lines represent the EQE_max_ with the photon recycling effect and unity IQE (i.e., *η*_inj_ = *η*_rad_ = 100%). b) Depth (*z*)‐profile of internal radiation which is finally outcoupled (i.e., relative contribution to EQE_max_), for LEDs with 10 nm‐thick (grey), 30 nm‐thick (red), 120 nm‐thick (green), and 200 nm‐thick (blue) perovskites. Position of the rear interface with TFB is defined to be zero. Inset represents the schematic of interference. c) The relative internal angular dipole intensity in perovskites LED with different emissive layer thickness (top) and relative intensity of horizontal dipole (*D_x_*) over vertical dipole (*D_z_*) monochromatically calculated in a single film (*N_s_* = 2.55 + 0.068*j* at 800 nm). d) Calculated LEE (= EQE / *η*_inj_ *η*_rad_) as a function of internal radiation efficiency (*η*_rad_). (dashed line: ray‐optics limit of 1/2*n* ^2^) e) EQE_max_ for the LED at a single wavelength of 800 nm with *n* = 2.55 and various *κ* values of the 200 nm‐thick perovskite emissive layer. The horizontal dashed line indicates the EQE_max_ = 27.6% from the full wavelength model. The vertical dotted line indicates *κ* = 0.068 of the FAPbI_3_ perovskite at the wavelength of 800 nm.

**Figure 5:**
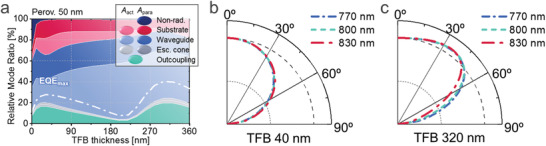
Variation of TFB optical spacer (between perovskite and MoO_3_/Au) thickness in a perovskite LED having a 50 nm‐thick FAPbI_3_ as an emissive layer. a) Relative ratio of the photon propagation through various modes as a function of TFB thickness. Relative external emission as a function of angle in the air mode for various wavelengths, for b) 40 nm and c) 320 nm‐thick TFB layers.

**Figure 6:**
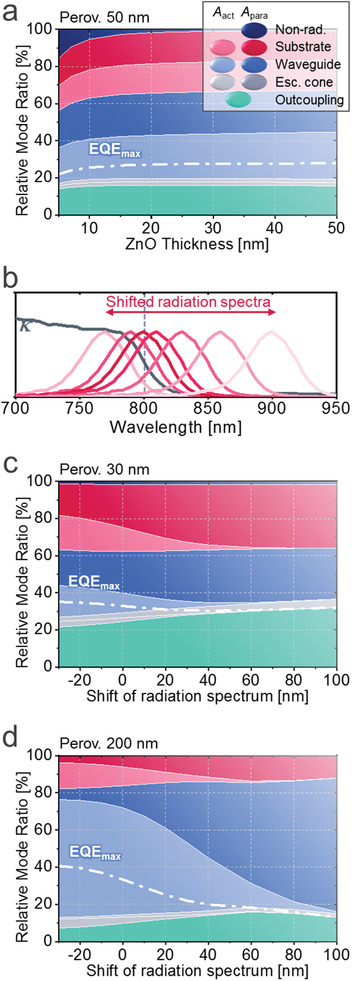
Calculated mode fractions and EQE_max_ for perovskite LEDs varying a) ZnO thickness and c,d) luminescence spectrum, depicted in (b). Perovskite thickness is 50, 30, 200 nm, for (a), (c), (d), respectively

**Figures** **4**–**6** show the results of various optical simulations based on the method described above (*M* = 20, *λ* = 720−860 nm), with a basic device structure^[^ ^2b^ ^]^ of glass/ ITO (150 nm)/ ZnO (30 nm)/ FAPbI_3_ perovskite (50 nm)/ TFB (40 nm)/ MoO_3_ (7 nm)/ Au (100 nm) unless otherwise specified. The fractions of power coupled into the different modes (outcoupling, absorption loss within escape cone, waveguide mode, substrate mode, and non‐radiative coupling) are calculated as a function of perovskite emissive layer thickness in Figure 4a. This analysis goes beyond the standard literature approaches^[^ ^2^, ^18^ ^]^ by including the effects of absorption in the emissive layer. This allows the absorption within the device to be separated into that absorbed within the emissive layer (*A*_act_), which can potentially be recycled, and that absorbed in other layers (*A*_para_). As shown in our previous paper,^[^ ^3c^ ^]^ as the perovskite gets thicker the fraction of direct outcoupling decreases but a high LEE is still possible with the aid of efficient photon recycling. Figure 4a also shows the maximum possible EQE value (EQE_max_), corresponding to *η*_rad_ = 100% and *η*_inj_ = 100%) as a function of perovskite thickness. While the direct outcoupling fraction is found to be highest (27.9%) for very thin perovskite (10 nm), the maxima in EQE_max_ occur at larger thicknesses (32.9% at 30 nm, and 33.4% at 200 nm).

Figure 4b provides further insight for the design of perovskite LED, showing distribution of the positions where outcoupled photons are generated within the perovskite layer, after recursive photon recycling with *η*_rad_ of 100%, for devices of various thickness. The outcoupling efficiency is determined almost entirely by the distance of the emissive dipole from the perovskite/TFB interface, and hence from the reflective gold electrode. The strong variation with dipole position is caused by optical interference between the directly emitted wave and the wave that is reflected from the gold electrode. Where constructive interference occurs for propagation normal to the device, light is coupled preferentially into the escape cone, whereas if destructive interference occurs for normal propagation light is coupled preferentially into non‐escape modes. The first resonant maximum occurs at ≈30 nm from the TFB, and a second maximum occurs at ≈190 nm. We note that the EQE_max_ peak in Figure 4a at 30 nm device thickness corresponds to most of the emission occurring close to an interference maximum, and the second EQE_max_ peak at 200 nm device thickness benefits from some emission occurring close to the second interference maximum. On the other hand, for devices with ≈120 nm thickness showing a low EQE_max_, the emission is weighted toward regions closer to the interference minimum. Although photon recycling can reclaim some photons from non‐escape modes, the presence of parasitic absorption in non‐emissive layers and non‐unity *η*_rad_ means that the interference effects described here still play a significant role in determining EQE. A reasonable estimate of the positions of the interference maxima can be obtained from the simple condition

(18) $$\sum n_{i}d_{i}=\left(\frac{1}{4}+\frac{N}{2}\right)\;\lambda$$

where *N* is an integer (0, 1, 2, ..) and Σ*n_i_d_i_* is the optical path length between the dipole and the back reflector. At *λ* = 800 nm, for 40 nm‐thick TFB (*n* = 1.57) and 7 nm‐thick MoO_3_ (*n* = 1.98) as spacers, Equation (18) is satisfied when the dipole is positioned at 48 nm (*N* = 0) or 205 nm (*N* = 1) from the interface between the perovskite (*n* = 2.55) and the TFB. The peaks in Figure 4b appear at slightly shorter distances due to the light penetration depth in the Au layer.

The top image of Figure 4c shows the internal angular distribution of dipole emission in perovskite LEDs of different thicknesses. Consistent with the discussion above, devices with optimal thicknesses of 30 nm and 200 nm show light concentrated into the escape cone, whereas for the 120 nm device the light is preferentially emitted into lateral modes. Interestingly, emission in the10 nm‐thick perovskite is more concentrated into the forward direction than in the 30 nm‐thick device, despite the poorer overlap with the interference maximum. This result can be attributed to the suppression of the rate of vertical dipole emission (mostly emitting in the lateral direction) due to optical confinement in the thin film with large refractive index (*n* ≈ 2.5 for FAPbI_3_ perovskite) enclosed by lower refractive index cladding materials (*n* < 1.6 for ZnO and TFB). In the pure film simulation with *N_s_* = 2.55 + 0.068*j* at *λ* = 800 nm, the relative emission rate of *x*‐oriented (horizontal) dipoles over the *z*‐oriented (vertical) dipoles was calculated to be 22.3 for a 10 nm‐thick film, much larger than 17.5 for 30 nm‐thick and 1.60 for 200 nm‐thick films, as shown in the bottom image. Where different emission orientations compete for the same pool of recombining carriers, this leads to emission being weighted toward horizontal dipole orientations. This effect results in the highest direct outcoupling ratio for 10 nm‐thick perovskite in Figure 4a, although its EQE_max_ is limited by insufficient photon recycling. The effect of dipole orientation on LEE shows the great potential of 2D perovskites to achieve high LED efficiencies surpassing those of 3D perovskites in the future.

Figure 4d shows the calculated LEE ( = EQE/IQE) with photon recycling, as a function of *η*_rad_. Unlike conventional non‐absorbing emitters with a constant LEE (i.e., with EQE linearly proportional to IQE), photon recycling makes LEE dependent on *η*_rad_. This dependence is particularly significant in thicker perovskites, due to the large fraction of *A*_act_ and the correspondingly large contribution of photon recycling. While LEE for 30 nm perovskite is more than twice of that for 200 nm at low *η*_rad_, the difference decreases as *η*_rad_ increases and they crossed at *η*_rad_ near 100%. For both thin and thick perovskites, the classical ray‐optics limit (1/2*n* ^2^) of outcoupling efficiency^[^ ^19^ ^]^ does not provide a useful measure when *η*_rad_ is high. While the thin (<100 nm) perovskites closer to the first optimal peak in Figure 4a (i.e., 30 nm) seem to be more popular in the currently reported perovskite LEDs,^[^ ^2^, ^3^, ^20^ ^]^ Figure 4d implies that thicker perovskites^[^ ^2c^ ^]^ also have the potential to achieve high efficiency along with the development of highly luminescent materials in the future.

Since the luminescence spectrum is typically narrow, previous optical studies for LEDs have mostly used a monochromatic model at the peak wavelength.^[^ ^2^, ^18^ ^]^ However, when absorption and emission spectra are overlapped as in perovskite, the refractive index (*n*+*jκ*) of the emissive material, especially the imaginary part (*κ*), varies rapidly even within the narrow emission spectrum. Figure 4e illustrates the deficiencies in the monochromatic model (wavelength of 800 nm, *N* = 2.55 + 0.068*j*). Compared to the full wavelength model of Figure 4a, the monochromatic model is shown to overestimate the EQE_max_ from 27.6% to 38.8%. The effective *κ* value required in the monochromatic model to give the correct result is shown to be 0.018, much lower than the value (0.068) at the PL peak wavelength. This shows the difficulty of applying the monochromatic model to perovskite LEDs when the effective *κ* is unknown.

Instead of changing emissive layer thickness, interference effects can be also optimized by changing optical spacers. Figure 5a shows the change of optical modes and EQE_max_ as a function of TFB thickness between perovskite and MoO_3_/Au. The first peak in EQE_max_ of 27.6% appears at 40 nm and the second peak appears at 320 nm with higher EQE_max_ of 39.9%. While currently reported perovskite LEDs mostly adopt thin spacers at the first peak, extension to a thicker spacer having sufficient conductivity would provide an opportunity for further breakthroughs.

Figure 5b,c shows the angular and spectral characteristics of perovskite LEDs. While the device with 40 nm‐thick TFB shows Lambertian‐like emission with little dependence on wavelength, that with 320 nm TFB shows distorted angular characteristics, which vary depending on the wavelength within the spectral range of emission. Such distortion occurs because the condition for optical interference is sensitive even to small variations of *n_i_*, *d_i_*, and *λ* in Equation (18), when the optical length is long (*N* ≥ 1). In display and lighting applications it is important to consider the angular distribution of light alongside optimizing efficiency measures for forward emission.

It is interesting that both the direct outcoupling and EQE_max_ modelled in our FAPbI_3_‐based device are higher than those for the recently reported 2D‐3D mixture PEA_2_Cs*_n_*_‐1_Pb*_n_*Br_3_ *_n_*_+1_ perovskite based LED (*n*_perov_ ≈ 2, emission near 520 nm, EQE_max_ ≈ 20%),^[^ ^3c^ ^]^ despite the relatively large refractive index (*n*_perov_ ≈ 2.5) limiting the angle of the escape cone. This can be mainly attributed to i) the short distance from perovskite to ITO (10 nm) in the reported device,^[^ ^3c^ ^]^ causing plasmon loss in ITO when the perovskite is thin; and ii) the smaller Stokes shift and higher re‐absorption coefficient in FAPbI_3_, increasing photon recycling due to the enhanced ratio of *A*_act_/*A*_para_. Regarding (i), overall plasmon loss (non‐radiative *A*_para_) increases from 1.0% to 5.3% when the ZnO thickness is changed from 30 to 10 nm in a FAPbI_3_ LED, as shown in Figure 6a. While previous studies on plasmon loss have mostly focused on back metal electrodes, the result shows that the loss through ITO also plays an important role and separation of ITO from the emissive layer needs to be taken into account for the design of efficient LEDs.

The influence of Stokes shift mentioned in (ii) can provide an insight for materials engineering. Figure 6c,d show the calculated mode fractions and EQE_max_ of LEDs with 30 nm‐thick and 200 nm‐thick FAPbI_3_ perovskites, respectively, on shifting the spectrum of internal radiation as represented in Figure 6b. Perovskite reabsorption (*A*_act_) decreases as PL is red‐shifted and Stokes shift increases. This results in the increase of direct outcoupling and a decrease of EQE_max_, and both efficiencies eventually converge to the same value. On the other hand, when radiation is blue‐shifted and anti‐Stokes shift is achieved,^[^ ^16^, ^21^ ^]^ high EQE_max_ is achieved with the maximized benefit of photon recycling despite low direct outcoupling efficiency. In case of large Stokes shift (100 nm), thin (30 nm) perovskite shows an outcoupling efficiency of 32.0%, higher than the 13.2% value for thick (200 nm) perovskite, owing to the aforementioned effects of dipole selection in due to interference effects. On the other hand, the highest EQE_max_ of 40.7% is shown for 200 nm‐thick perovskite with the largest reabsorption (PL shift of −30 nm), clearly above the 35.1% seen for 30 nm‐thick perovskite. Small Stokes shift is a distinct optical property of perovskites, contrasting with existing organic emitters typically having shifts of around 100 nm. The results show the potential of perovskite LEDs, implying that there remains substantial room for enhancement in the currently reported EQEs near 20%.

## 8 Conclusion

We have investigated the optical modelling methods for perovskite LEDs, having re‐absorption and re‐emission properties. TMF is used to calculate the Poynting vectors at each interface and non‐radiative emitter‐coupling is excluded to avoid divergence. Photon recycling is taken into account by the recursive calculation of the dipole generation profile. Resulting EQE_max_ with photon recycling is shown to be clearly above the direct outcoupling ratio. The effects of microcavity, dipole orientation, and photon recycling have been investigated for the future design of efficient perovskite LEDs. In addition to perovskite LEDs, application of the proposed method can be also expanded to various thin‐film semiconductors (organics, nanocrystals, etc.) having small Stokes shift or various optoelectronic devices (photovoltaics, lasers, etc.) where photon radiation plays an important role.

## Conflict of Interest

N.G. is a founder of a company commercializing perovskite emitters.
